# The role of integration in oncogenic progression of HPV-associated cancers

**DOI:** 10.1371/journal.ppat.1006211

**Published:** 2017-04-06

**Authors:** Alison A. McBride, Alix Warburton

**Affiliations:** Laboratory of Viral Diseases, National Institute of Allergy and Infectious Diseases, National Institutes of Health, Bethesda, Maryland, United States of America; University of Michigan Medical School, UNITED STATES

## Introduction

Persistent infection with a subset of “high oncogenic risk” human papillomaviruses (HPVs) can promote the development of cancer. In these cancers, the extrachromosomal viral genome has often become integrated into the host genome. The integration event is thought to drive oncogenesis by dysregulating expression of the E6 and E7 viral oncogenes, leading to inactivation of critical cell cycle checkpoints and increased genetic instability in the host. This Pearl reviews the evidence that gave rise to the current textbook paradigm of HPV integration events and their consequences and incorporates new findings that demonstrate that stochastic integration events can promote oncogenesis in many ways.

## Is HPV integration part of the papillomavirus life cycle?

Papillomaviruses have a resourceful life cycle that takes advantage of the tissue renewal process of stratified epithelia. Only the lower, basal cells in the epithelium proliferate, but they can divide either symmetrically (to produce more basal cells) or asymmetrically (one of the daughter cells leaves the basal layer and begins the differentiation process). Differentiating daughter cells move up through the epithelium, acquiring specialized properties until they are released from the surface as part of the process of tissue renewal. Papillomaviruses exploit this process; they access and infect the basal cells through a micro-abrasion and establish a long-term infection in these dividing cells. The viral E1 and E2 proteins support replication of the viral genome at a low copy number in the basal cells as a small, dsDNA nuclear plasmid of about 7–8 kbp. It is only when these infected cells differentiate and move towards the surface of the epithelium that high levels of viral DNA are synthesized, packaged in virions, and sloughed from the surface of the epithelium in viral-laden squames [1]. HPVs are often found integrated in premalignant lesions and a range of anogenital and oropharyngeal cancers [2–4], but this is not part of the viral life cycle. In fact, integration is a dead end for the virus, as it is no longer able to form a small, circular genome that can be packaged and transmitted to a new host.

## How does integration of HPV promote oncogenesis?

Almost all HPV integration events that have been studied in detail to date are related to HPV oncogenesis. HPV integration events can be detected in premalignant lesions, but the percentage of cells containing integrated HPV increases as cells progress to invasive cancer [5]. Integration usually results in dysregulation of expression of the viral E6 and E7 oncogenes, which promotes cellular proliferation, abrogates cell cycle checkpoints, and causes progressive genetic instability. This gives cells a selective growth advantage and promotes oncogenic progression [6]. Clonal outgrowth of cells with integrated HPV highlights the importance of dysregulated oncogene expression. In fact, HPV-associated cancers are dependent on the expression of the viral E6 and E7 oncogenes for continued proliferation and survival [7].

**Fig 1:**
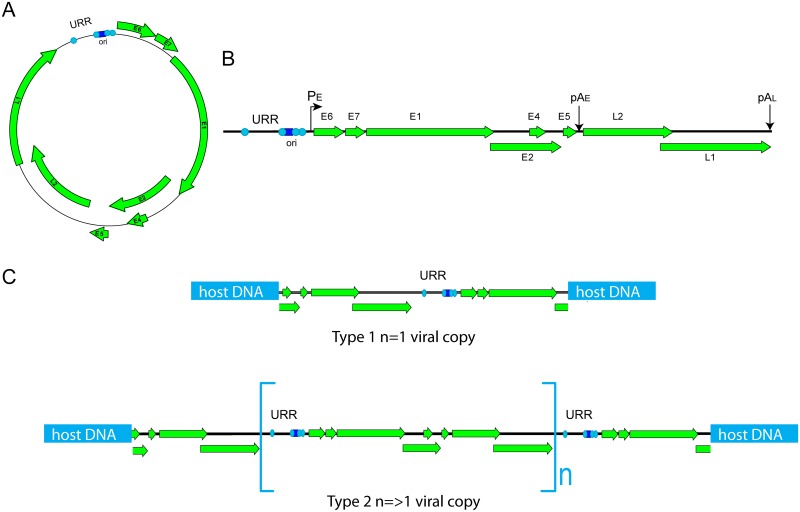
Types of HPV integration. A. Circular HPV genome. B. Linear HPV genome. URR (upstream regulatory region), P_E_ (early promoter), and pA_E_ and pA_L_ (early and late polyadenylation sites) are indicated. The light blue circles in the URR represent E2 binding sites, and the dark blue square is the E1 binding site in the origin of replication (ori). C. In Type 1 integration, a single viral genome is integrated into the host DNA. In Type 2 integration, multiple genomes are integrated in tandem in a head-to-tail orientation. This often is accompanied by focal rearrangement and amplification of flanking cellular sequences.

HPV integration can be classified into two types: in Type 1, a single genome is integrated into cellular DNA; and in Type 2, multiple tandem head-to-tail repeats of the genome, in some cases with intervening cellular flanking sequences, are found at a single genomic locus [6] (see Fig 1). In both types, RNA encoding the E6 and E7 oncogenes is initiated from the major promoter in the viral upstream regulatory region (URR) and is spliced from a splice donor in the viral genome to a splice acceptor in the host DNA [8]. In Type 2 integration, there is evidence that usually only the 3’ junctional copy of the viral genome is transcriptionally active [9]. Continued expression of the E1 and E2 replication proteins from integrated genomes causes focal genomic instability at the integration locus [10, 11], and in most cases, when complete genomes are tandemly integrated, the internal copies are silenced by DNA methylation [12].

**Fig 2:**
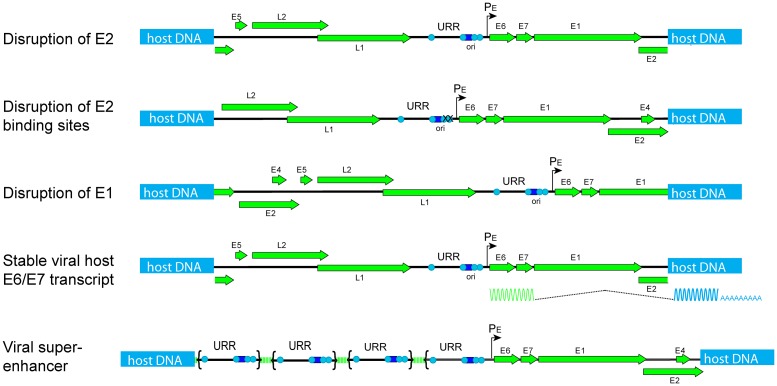
Models of integration events that promote oncogenesis. The five integration models shown in Table 1 are shown in the diagram, as indicated to the left. URR (upstream regulatory region), and P_E_ (early promoter), are indicated. The light blue circles in the URR represent E2 binding sites, and the dark blue square is the E1 binding site in the origin of replication (ori).

Viral genome integration events usually result in dysregulation of E6 and E7 gene expression compared to that expressed from extrachromosomal viral genomes, and this can be achieved in a number of ways (see Fig 2 and Table 1). The earliest model proposed that the integration event disrupts the E2 gene, alleviating E2 transcriptional repression of the E6 and E7 promoter and thus driving oncogene expression [13]. E2 regulation can also be disrupted by methylation of the E2 binding sites in the URR [14]. E6/E7 oncogene expression can also be modulated by epigenetic events that do not directly affect E2 DNA binding. Our group recently demonstrated that tandemly integrated repeats of HPV16 DNA could develop into a Brd4-dependent super-enhancer to drive strong expression of the viral oncogenes [15]. Viral genomes are also often integrated in such a way as to disrupt the gene that encodes the E1 replicative helicase, which also disrupts the downstream E2 gene. In HPV infection, the level and nuclear location of the E1 protein are tightly regulated because uncontrolled E1 expression can cause DNA damage and growth arrest [16, 17]. E1 expression can also promote focal genomic instability by inducing overamplification of the integration region [10, 11]. Thus, disruption of the E1 gene could give a selective growth advantage and promote clonal expansion. As mentioned above, most integration events result in expression of a spliced viral—cellular transcript. Jeon and Lambert demonstrated that these fusion transcripts are very often more stable than their viral counterparts, yet again increasing HPV oncogene expression [18]. There are also cases in which an oncogenic HPV has integrated in the vicinity of a cellular oncogene or tumor suppressor gene [2, 19], but this is not thought to be a universal way in which HPV promotes oncogenesis.

**Table 1:** Integration events that promote oncogenesis.

Integration event	Effect on oncogenic progression
Disruption of E2 gene	Abrogation of E2-mediated transcriptional repression of the E6/E7 promoter
Disruption of E2 binding sites by methylation	Abrogation of E2-mediated transcriptional repression of the E6/E7 promoter
Disruption of E1 gene	Abrogation of E1 replication activities that can induce DNA damage and growth arrest and promote focal genomic instability at the integration locus
Formation of viral—host fusion transcript	Hybrid E6/E7 transcripts are often more stable than viral E6/E7 transcripts
Tandem repeats of HPV genome/regulatory elements	Internal copies are usually silenced, but sometimes tandem copies of active genomes can function as a transcriptional super-enhancer
Altered regulation of cancer-associated genes in the vicinity of the integration locus	Integrated HPV can disrupt cellular genes and/or their flanking sequences, altering their expression and/or the expression of nearby genes; infrequently, HPV integration alters the expression of nearby cancer genes, thus promoting carcinogenesis

## Do all HPV-associated cancers contain integrated HPV DNA?

Although many HPV-associated cancers contain integrated viral DNA, it is not universal. HPV-associated cancers can contain either integrated HPV DNA, extrachromosomal viral DNA, or a mix of both [20]. However, in tumors with exclusively extrachromosomal viral DNA, the viral genome has usually acquired genetic or epigenetic changes that result in dysregulated E6/E7 gene expression [12, 21, 22]. For example, methylation of the E2 binding sites in the URR can alleviate E2-mediated repression of the viral oncogenes [22–24]. Therefore, while integration is very frequently detected in HPV-associated cancers, it is not absolutely required.

Analysis of samples from The Cancer Genome Atlas study shows that HPV integration occurs in >80% of HPV-positive cervical cancers [25]. Of these, 76% of HPV16-positive samples have integrated HPV, whereas integration is evident in all HPV18-positive samples. This confirms early observations of different frequencies of integration between HPV16 and HPV18 [26]. In HPV-positive oropharyngeal squamous cell carcinomas, the incidence of viral integration is lower, and many tumors have either extrachromosomal or mixed extrachromosomal and integrated viral DNA [2, 27–29]. The rate of HPV integration in other anogenital cancers is not as well documented. One study reports that almost 80% of anal carcinomas contain integrated HPV; however, the vast majority of these samples also contain extrachromosomal genomes [30]. Many different methods have been used to detect integrated viral genomes, and more research is needed to clarify the significance of the observed differential rates of integration and to determine whether HPVs promote oncogenesis by different mechanisms at different anatomical locations.

## Are there specific integration target sequences in the human and viral genomes?

Genome-wide efforts to elucidate a genomic signature of HPV integration events have identified only a handful of recurrent loci. These so-called genomic “hotspots” are highly correlated with common fragile sites [31] and transcriptionally active regions of the genome [19, 32]. Regions of microhomology (1–10 bp) between viral and human genomic sequences are sometimes found at integration breakpoints [2], as have AT-rich regions of the genome that have the potential to form stem—loop structures that promote the formation of stalled replication forks during replication stress [28]. There are also examples of HPV integration resulting in insertional mutagenesis and/or potential regulatory effects on neighbouring genes [33]. Increased integration within cancer-associated genes or pathways, including the MYC gene locus, have also been reported [2, 34]. However, this is not a universal phenomenon associated with HPV integration [4].

The best-characterized HPV integration sites are those responsible for HPV cervical cancers. In these sites, the E6 and E7 genes are invariably expressed from viral promoters in the URR, and the viral genome is most often disrupted in the E1 and E2 genes, leading to the models described above. With the advent of more sensitive techniques, it is becoming apparent that there are often multiple integration sites, and many are disrupted at positions throughout the viral genome [35]. However, it is likely that these are background/silent integration events [36], resulting from overall increased genetic instability, and that most of these genomes are passengers rather than drivers of oncogenesis. Likely, non-oncogenic HPVs also occasionally become integrated, but without oncogene-driven clonal expansion, these rare events are almost never detected.

It is becoming clear that there is very often rearrangement and amplification of cellular sequences flanking the HPV integration site [2, 35, 37]. The initial integration event likely occurs in a cell that also harbors extrachromosomal genomes that express the E1 and E2 replication proteins. In this scenario, the E1 and E2 proteins cause overamplification, or onion skin replication, of the integrated HPV genome and adjacent sequences [10, 11]. This results in heterogeneous replication intermediates that serve as substrates for recombination and repair, resulting in rearrangements, deletions, and amplification of viral and host sequences [10, 11]. A looping model also describes how viral—host DNA concatemers can arise from the formation of a transient loop that acts as a substrate for rolling circle replication [35]. Additional rearrangements could be further exacerbated by the inherent genomic instability of the host region (e.g., common fragile sites), from the instability of the tandemly repeated locus, and from continuing E6/E7-mediated genetic instability.

## What promotes HPV integration?

There are several stages in the development of an HPV integration site that eventually drives oncogenic progression. The initial integration event likely takes place in regions where the viral and host DNA are in close proximity in the proliferating basal cells of a lesion. HPV hijacks the host DNA damage response to replicate its own DNA in certain phases of the life cycle [26, 27], and this occurs adjacent to regions of the host DNA susceptible to replication stress (e.g., common fragile sites) [38]. This could explain the preferential integration in these regions [28]. Secondly, the target region must be in a transcriptionally competent region of host chromatin that can support viral oncogene expression. In most cases, E6/E7 mRNA is expressed as a viral—host fusion transcript, and this necessitates the presence of a nearby cellular splice acceptor and polyadenylation site (often cryptic). Finally, it is likely that there is epigenetic modulation of the integration site (DNA methylation and chromatin modifications) that further determine whether the integration site is active or silenced [12, 39]. Therefore, many events and processes contribute to the development of an HPV integration event that is a strong driver of oncogenesis; there are likely many dead-end integration events that fail to produce sufficient E6/E7 oncoproteins to drive clonal expansion of the host cell.
